# Regioselective Synthesis of Procyanidin B6, A 4-6-Condensed (+)-Catechin Dimer, by Intramolecular Condensation

**DOI:** 10.3390/molecules23010205

**Published:** 2018-01-18

**Authors:** Yusuke Higashino, Taisuke Okamoto, Kazuki Mori, Takashi Kawasaki, Masahiro Hamada, Noriyuki Nakajima, Akiko Saito

**Affiliations:** 1 Graduate School of Engineering, Osaka Electro-Communication University (OECU), 18-8 Hatsu-cho, Neyagawa-shi, Osaka 572-8530, Japan; de15a002@oecu.jp (Y.H.); me12a004@osakac.info (T.O.); me14a009@oecu.jp (K.M.); 2 College of Pharmaceutical Sciences, Ritsumeikan University, 1-1-1 Nojihigashi, Kusatsu, Shiga 525-8577, Japan; kawa0227@fc.ritsumei.ac.jp; 3 Department of Pharmaceutical Engineering, Faculty of Engineering, Toyama Prefectural University (TPU), 5180, Kurokawa, Imizu, Toyama 939-0398, Japan; hamada@pu-toyama.ac.jp (M.H.); nori@pu-toyama.ac.jp (N.N.)

**Keywords:** condensed tannin, proanthocyanidins, intramolecular condensation, procyanidin B6

## Abstract

Proanthocyanidins, also known as condensed tannins or oligomeric flavonoids, are found in many edible plants and exhibit interesting biological activities. Herein, we report a new, simple method for the stereoselective synthesis of procyanidin B6, a (+)-catechin-(4-6)-(+)-catechin dimer, by Lewis acid-catalyzed intramolecular condensation. The 5-*O*-*t*-butyldimethylsilyl (TBDMS) group of 5,7,3′4′-tetra-*O*-TBDMS-(+)-catechin was regioselectively removed using trifluoroacetic acid, leading to the “regio-controlled” synthesis of procyanidin B6. The 5-hydroxyl group of the 7,3′,4′-tri-*O*-TBDMS-(+)-catechin nucleophile and the 3-hydroxyl group of 5,7,3′,4′-tetra-*O*-benzylated-(+)-catechin electrophile were connected with an azelaic acid. The subsequent SnCl_4_-catalyzed intramolecular condensation proceeded smoothly to give the 4-6-condensed catechin dimer. This is the first report on the complete regioselective synthesis of a 4-6-connected oligomer without modifying the 8-position.

## 1. Introduction

**Figure 1:**
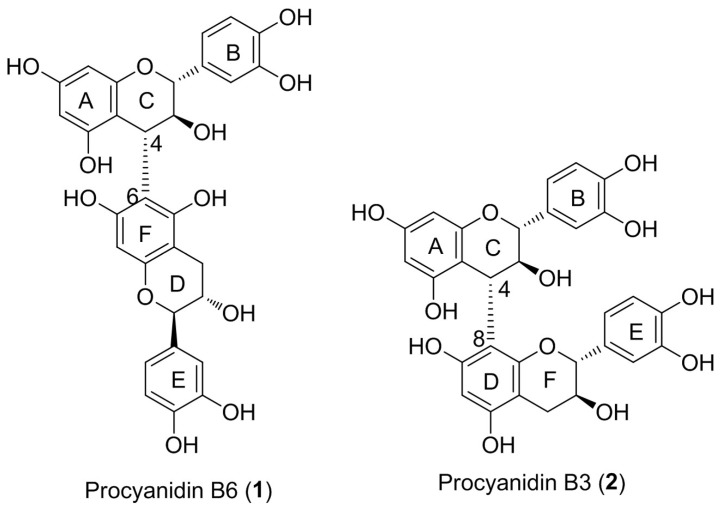
Structures of procyanidin B6 (**1**) and procyanidin B3 (**2**).

Proanthocyanidins are a class of polyphenols and are generally referred to as condensed tannins or oligomeric flavonoids. They have attracted a great deal of attention because of their strong antioxidant activity and wide range of interesting biological functions [1,2]. However, proanthocyanidins are often obtained as a mixture of various analogues from plants. Thus, despite their simple structure as the derivatives of flavan-3-ols, the purification of each compound remains difficult. Flavan-3-ols such as catechin and epicatechin are one of the most well-known groups of biologically active polyphenols. The oligomers composed of these catechins and epicatechin are known as highly functional polyphenols contained in familiar foods such as cacao [3,4]. There are many reports related to other biological activities, such as antifungal [5,6], antiviral [5,7], anti-inflammatory [8], anticancer [3,9], and treating heart diseases [10], among others. Flavan-3-ol derivatives including proanthocyanidins are also converted to various structurally complex secondary polyphenols through chemical reactions that occur during food processing such as drying [11], frozen storage [12], and acidic treatment [13]. These complex and diverse forms make the isolation, structural determination, and evaluation of the biological activities of each compound much more difficult. Therefore, securing sufficient amount of proanthocyanidin as a pure compound for experiment is also important for elucidating the function of proanthocyanidin. A synthetic route to oligomeric catechin and epicatechin derivatives has been developed by Kozikowski et al. [14,15,16] and subsequently by other research groups [17,18,19,20,21,22,23,24]. Additionally, our group has developed and reported simple and stereoselective methods to synthesize 4-8 condensed procyanidin oligomers (constituted by flavan-3-ols having two hydroxyl groups on B and E rings) [25,26,27,28,29,30,31,32,33,34,35,36,37,38] and prodelphinidin oligomers (constituted by flavan-3-ols having, three hydroxyl groups on B and/or E rings) [39] and then studied their biological activities. The key step of the synthesis of proanthocyanidins is the coupling reaction between the nucleophile and electrophile using a Lewis acid such as TiCl_4_, SnCl_4_, or trimethylsilyl triflate (TMSOTf) as an activator. As shown in Figure 1, the two dimers of (+)-catechin, 4-6-condensed procyanidin B6 (**1**) and 4-8-condensed procyanidin B3 (**2**), are isomers with different bonding positions. The abundance of the 4-6-linked oligomer is comparatively low. In particular, the biological activities of various 4-6-condensed oligomers are yet to be studied in detail due to the unavailability of these compounds. Most studies on the synthesis of proanthocyanidins focus on 4-8-linked oligomers as target compounds, and only a few of them address the stereoselective synthesis of 4-6-linked oligomers. The cool synthesis of the 4-6 catechin dimer, procyanidin B6 (**1**), has been previously achieved by combining the 8-halo-capping strategy [24].

Due to increasing interest toward the structure-activity-relationship studies (SAR) of proanthocyanidins, developing new and simple methods for the synthesis of various types of proanthocyanidins has become increasingly important. Therefore, we focused on developing a very simple approach to the selective synthesis of such compounds. Herein, we report on an easy, applicable, and reliable method for the stereoselective synthesis of procyanidin B6 (**1**) via an intramolecular condensation reaction without modifying the 8-position.

## 2. Results and Discussion

**Scheme 1:**
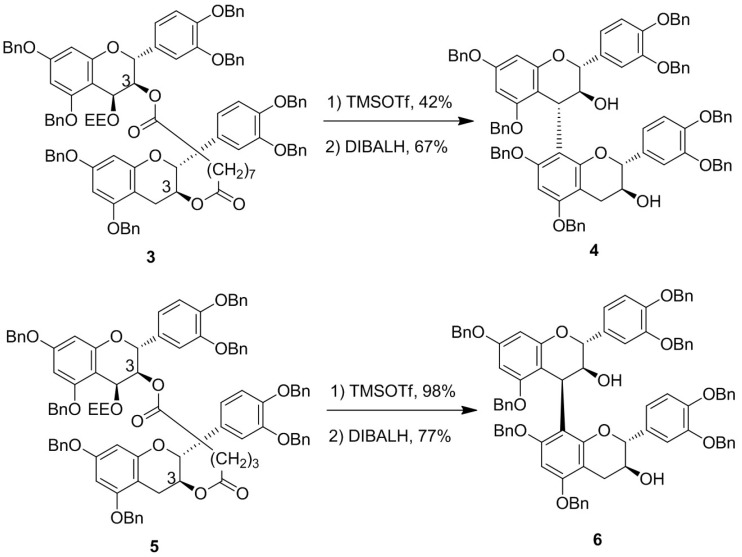
Stereoselective synthesis of the basic skeleton of 3,4-*trans* and 3,4*-cis*-(+)-catechin dimers via an intramolecular coupling.

Previously, we developed a Lewis acid-catalyzed “intramolecular” one-to-one coupling approach that allows for the stereoselective synthesis of 4-8-condensed (+)-catechin dimers (**4**, **6**: Scheme 1) [28,31]. The method includes the connection of flavan-3-ol-derived nucleophile and electrophile via a diester linker and the subsequent condensation reaction by “intramolecular” coupling. Furthermore, the stereochemistry of the coupling product was found to depend on the length of the linker. Despite the fact that the 3,4-*cis* structure is not preferentially obtained in intermolecular reactions, the intramolecular TMSOTf-catalyzed coupling of azelaic acid (C9 dicarboxylic acid) diester (**3**) afforded 3,4-*trans* product **4**, and the coupling of glutaryl (C3 dicarboxylic acid derivative) diester (**5**) gave 3,4-*cis* product **6** [28] (Scheme 1). Therefore, this intramolecular coupling method could potentially solve the difficulties surrounding the stereoselective synthesis of proanthocyanidins such as 3,4-*cis* oligomers. Additionally, it could be possible that the structure of the intramolecular coupling product is changed depending on the position in which the diester linker is introduced. Following these assumptions, we attempted to introduce a diester linker regioselectively to another position by selectively deprotecting one of the four protecting groups of the phenolic hydroxyl moieties, which are in similar environments.

**Scheme 2:**
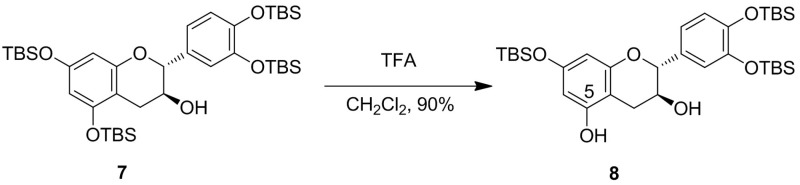
Regioselective deprotection of the 5-*O*-*t*-butyldimethylsilyl (TBDMS) group.

A thorough examination of the reactive properties of the protected flavan-3-ols revealed that the 5-*O*-TBDMS (TBS) group of the 5,7,3′,4′-tetra-*O*-TBDMS-(+)-catechin (**7**) could be regioselectively removed with TFA to give **8** in 90% yield [40] (Scheme 2). The structure of the 5-OH product (**8**) was confirmed by the HMBC (Heteronuclear Multiple Bond Correlation) between C5 and the hydroxyl proton of **8** [40].

**Scheme 3:**
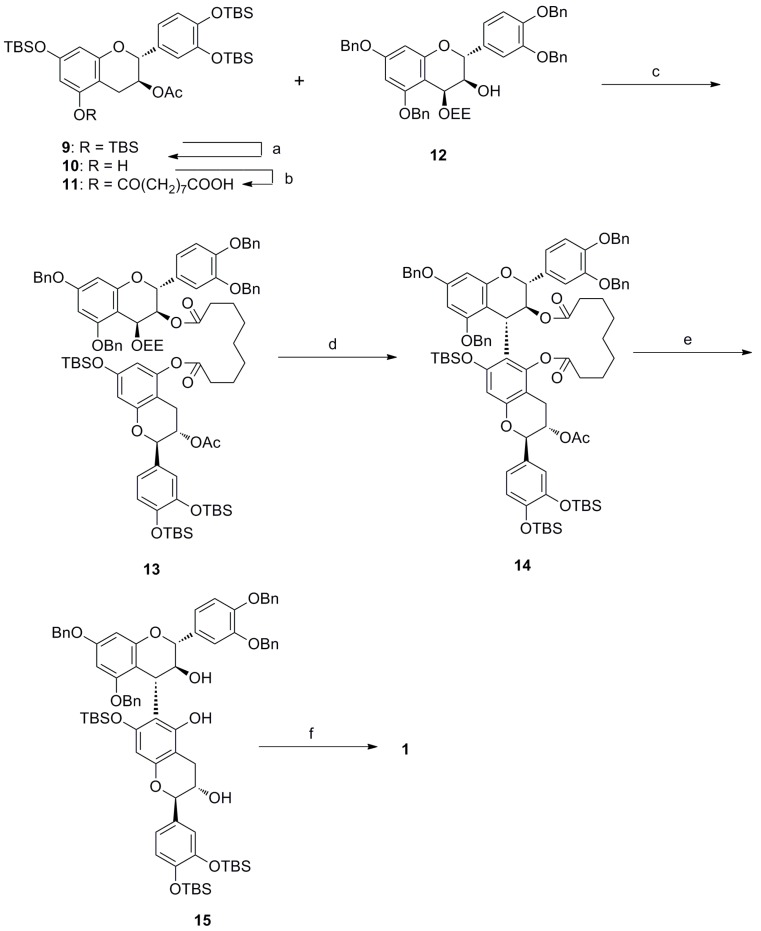
Stereoselective synthesis of the procyanidin B6 (**1**). Reaction conditions: (a) TFA, CH_2_Cl_2_; 76% yield; (b) Azelaic acid, DCC, DMAP, CH_2_Cl_2_; 80% yield; (c) DCC, DMAP, CH_2_Cl_2_; 58% yield; (d) SnCl_4_, CH_2_Cl_2_, −20 °C; 72% yield; (e) DIBALH, CH_2_Cl_2_; 66% yield; (f) (1) TBAF, AcOH, THF, r.t.; 85% yield; (2) Pd(OH)_2_/C, H_2_, THF/MeOH/H_2_O (20/1/1); 65% yield.

**Figure 2:**
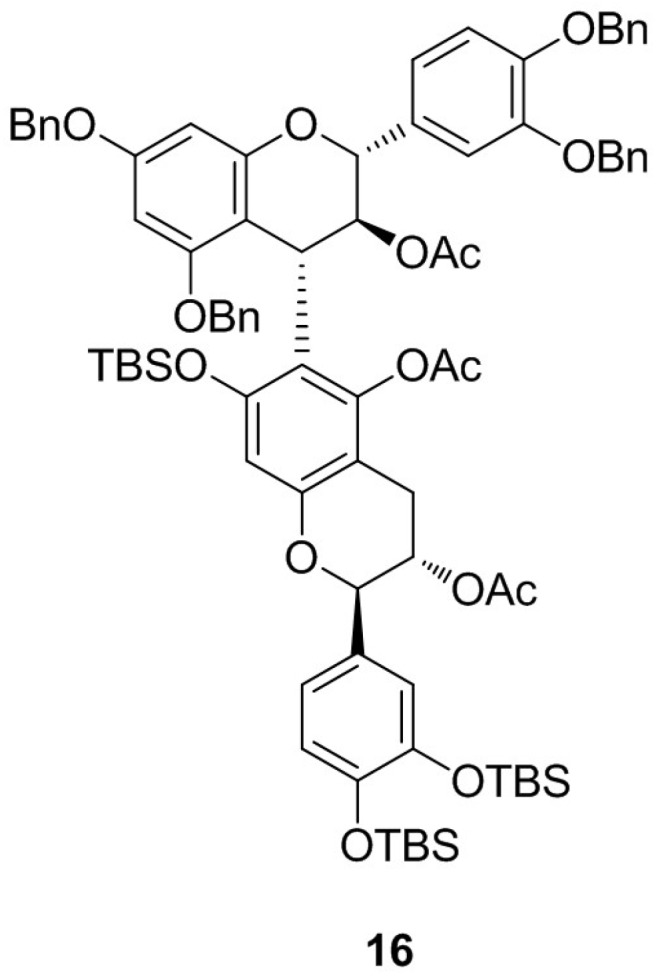
Acetylation derivative **16**.

Using the aforementioned regioselective deprotection method, the intramolecular synthesis of the 4-6-condensed procyanidin B6 (**1**) was achieved (Scheme 3). During this synthesis, the 5-*O*-TBDMS group of an acetylation derivative of **7** (**9**) was removed with TFA to give 5-hydroxyl nucleophile **10** in 76% yield. The 5-hydroxyl group of **10** was then esterified with azelaic acid using the *N*,*N*′-dicyclohexylcarbodiimide (DCC) condensation method to afford the carboxylic acid derivative **11** in 80% yield. The 3-hydroxyl group of **12** (electrophile) [26] was then connected to **11** by esterification to obtain the diester compound **13**. The subsequent SnCl_4_-catalyzed intramolecular condensation of **13** at −20 °C proceeded smoothly to afford the 4-6 condensed catechin dimer **14** in 72% yield. When the cyclization reaction was carried out with TMSOTf instead, another TBDMS group was also deprotected, resulting in a complicated mixture of many byproducts. Additionally, the use of dicarboxylic acids as linkers was unsuccessful, and the intramolecular cyclization proceeded only when azelaic acid was employed. Using linkers of other lengths led to self-condensation products of electrophile **12**, which once again caused the formation of a complicated mixture of byproducts. Temperature optimization studies of the cyclization reaction showed that the yield was the highest when the reaction was carried out at −20 °C. A decrease in the yield was observed both at higher and lower temperatures. The subsequent removal of the diester linker in **14** via diisobutylaluminum hydrate (DIBAL) reduction gave triol **15** in 66% yield. Due to peak broadening in the NMR spectrum of **15**, the tri-acetylated compound **16** was prepared in order to determine the dimeric structure and 3,4-*trans* stereochemistry of the synthesized product (Figure 2). Compound **15** was eventually converted to 3,4-*trans*-(+)-catechin-(4-6)-(+)-catechin dimer, procyanidin B6 (**1**), after TBDMS-group deprotection by tetrabutylammonium fluoride (TBAF) (85% yield) and subsequent Pd(OH)_2_-catalyzed hydrogenolysis (65% yield). The full spectroscopic data confirmed the structure of procyanidin B6 (**1**) [41].

**Scheme 4:**
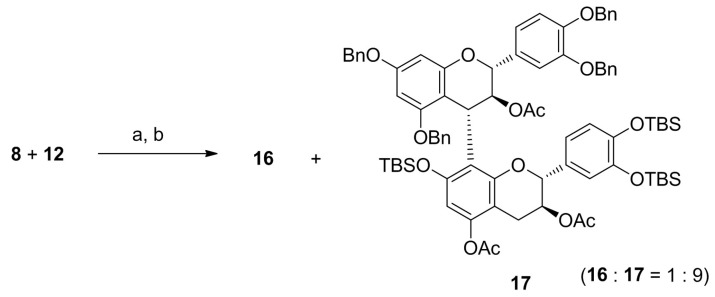
Intermolecular coupling of **8** and **12**. Reaction conditions: (a) TMSOTf, CH_2_Cl_2_, −40 °C; 78% yield; (b) Acetic anhydride, DMAP, pyridine, quant.

In addition, the coupling reaction of nucleophile **8** and electrophile **12** led to the formation of 4-6 and 4-8-condensed compounds (Scheme 4). After the intermolecular coupling products were acetylated, **16** and **17** were isolated and their structures were confirmed. Compound **16**, which was synthesized via intramolecular coupling, was identified with the compound resulting from the intermolecular coupling. Thus, the intramolecularly cyclized product is a 4-6-connected dimer. Usually, when the 5-position is protected, a 4-6-condensed compound cannot be obtained. Therefore, it is possible that the electrophile was brought close to the 6-position through 5-TBDMS group deprotection. However, reactivity at the 6-position was extremely low as compared to that at the 8-position, and the ratio of the 4-6 and 4-8-condensed products was 1:9.

## 3. Materials and Methods

All commercially available chemicals for the chemical syntheses were used without further purification. All reactions were performed under argon atmosphere and monitored with thin-layer chromatography (TLC) using 0.25 mm pre-coated silica gel plates 60F254 (Art 5715, Merck KGaA, Darmstadt, Germany). An ATAGO (Minato, Tokyo, Japan) AP-300 spectrometer was used to measure the optical rotation. ^1^H- and ^13^C-NMR spectra were recorded on an Agilent Inova 500 Spectrometer (500/125 MHz) and DD2 NMR Spectrometer (400/100 MHz) (Agilent, Santa Clara, CA, USA). A JEOL JMS-AX500 mass spectrometer was used to acquire the fast atom bombardment (FAB) mass spectra. A microTOFfocus mass spectrometer (Bruker Daltonics, Billerica, MA, USA) was used to acquire the electrospray ionization (ESI) mass spectra. High-performance liquid chromatography (HPLC) purifications were carried out on an Ascentis^®^ column (SUPELCO^®^ analytical, Sigma Aldrich Co., St. Louis, MO, USA; 25 cm × 21.5 mm, 5 μm) using the solvents (A) 0.05% HCOOH in CH_3_CN, and (B) 0.05% HCOOH and 10% CH_3_CN in H_2_O. Elution was performed with a linear gradient 20–100% B in 25 min (4.0 mL/min flow rate).

*3-Acetoxy-3*′*,4*′*,7-tri(t-butyldimethylsilyloxy)-(+)-catechin* (**10**). To a solution of **9** (0.27 g, 0.34 mmol) in CH_2_Cl_2_ (50 mL) was added TFA (0.031 mL, 0.41 mmol) slowly at 0 °C. After stirring for 6 h, the reaction mixture was quenched with sat. NaHCO_3_ solution in water. The aqueous solution was extracted with CHCl_3_, and the organic phase was washed with water and brine and then dried (MgSO_4_). Filtration, concentration, and silica-gel column purification (*n*-hexane/EtOAc, 10:1 to 3:1) afforded 0.18 g (0.26 mmol, 76%) of **10** as an amorphous solid. [α]${}_{D}^{24}$ +61.8 (*c* 0.21, CHCl_3_); ^1^H-NMR (500 MHz, CDCl_3_) 6.74 (1H, br), 6.71 (2H, br), 5.99 (1H, d, *J* = 2.0 Hz), 5.89 (1H, d, *J* = 2.0 Hz), 5.19 (1H, ddd, *J* = 5.5, 5.5, 6.0 Hz), 4.92 (1H, d, *J* = 5.5 Hz), 2.73 (1H, dd, *J* = 5.5, 7.0 Hz), 2.59 (1H, dd, *J* = 6.0, 17.0 Hz), 2.35 (1H, br), 1.88 (3H, s), 0.89–0.83 (27H, m), 0.11 (9H, s), 0.09 (3H, s), 0.05 (3H, s), 0.03 (3H, s); ^13^C-NMR (125 MHz, CDCl_3_) 170.5, 155.5, 155.0, 154.6, 147.0, 146.8, 131.0, 121.1, 119.4, 119.1, 100.6, 100.3, 100.0, 77.8, 69.3, 25.9 (2), 25.7, 23.1, 21.1, −4.06, −4.11, −4.15, −4.24, −4.40, −4.43; FABMS (*m*/*z*): 676 (12), 675 (M^+^ + H, 12), 674 (6), 617 (13), 616 (23), 615 (45); 614 (23), 613 (14), 558 (10), 557 (20), 444 (10), 443 (29), 368 (26), 367 (81), 351 (66), 277 (28), 254 (18), 253 (86), 205 (16), 179 (100); HRFABMS: Calcd. for C_35_H_59_O_7_Si_3_, 675.3569; Found, 675.3563.

*5-Azelaic acid ester* (**11**). To a solution of **10** (0.47 g, 0.70 mmol) and azelaic acid (0.39 g, 2.00 mmol) was added DCC (0.17 g, 0.83 mmol) and cat. DMAP in CH_2_Cl_2_ (50 mL) at 0 °C. After stirring for 24 h at r.t., the reaction mixture was quenched with water. The aqueous solution was extracted with CHCl_3_, and the organic phase was washed with water and brine and then dried (MgSO_4_). Filtration, concentration, and silica gel column purification (CHCl_3_/EtOAc, 200:1 to 2:1 followed by *n*-hexane/EtOAc, 10:1 to 1:1) afforded 0.47 g (1.60 mmol, 80%) of **11** as an amorphous solid. [α]${}_{D}^{24}$ +196.2 (*c* 0.50, CHCl_3_); ^1^H-NMR (400 MHz, CDCl_3_) 10.40–10.10 (1H, br), 6.80 (1H, d, *J* = 8.3 Hz), 6.80 (1H, 1.8 Hz), 6.77 (1H, dd, *J* = 1.8, 8.3 Hz), 6.36 (1H, d, *J* = 2.4 Hz), 6.23 (1H, d, *J* = 2.4 Hz), 5.23 (1H, ddd, *J* = 5.2, 6.3, 6.4 Hz), 2.75 (1H, dd, *J* = 5.2, 16.5 Hz), 2.56 (1H, dd, *J* = 6.4, 16.5 Hz), 2.51 (2H, t, *J* = 7.7 Hz), 2.35 (2H, t, *J* = 7.5 Hz), 1.95 (3H, s), 1.78–1.60 (4H, m), 1.43–1.30 (6H, m), 0.97 (9H, s), 0.96 (9H, s), 0.94 (9H, s), 0.20 (6H, s), 0.185 (3H, s), 0.175 (3H, s), 0.13 (3H, s), 0.12 (3H, s); ^13^C-NMR (100 MHz, CDCl_3_) 171.4, 171.3, 170.4, 155.3, 154.9, 149.9, 147.1, 147.0, 130.8, 121.2, 119.6, 119.3, 107.4, 105.7, 105.6, 78.2, 69.0, 34.3, 34.0, 29.02, 28.96, 28.94, 26.04, 26.03, 25.7, 24.9, 24.7, 23.9, 21.6, 18.6, 18.3, −3.95, −3.98, −4.05, −4.10, −4.32, −4.35; ESIMS (*m*/*z*) 1153 (61), 1152 (68), 1151 (80), 1076 (43), 1075 (79), 1074 (100), 869 (18), 868 (37), 687 ([M + Na]^+^, 56); ESIHRMS Calcd. for C_44_H_72_O_10_Si_3_Na, 867.4331; Found, 867.4340.

*Cyclization precursor* **13**. To a solution of **11** (0.34 g, 0.40 mmol) and **12** (0.77 g, 1.00 mmol) was added DCC (99.0 mg, 0.48 mmol) and catalytic amount of DMAP in CH_2_Cl_2_ (20 mL) at 0 °C. After stirring for 24 h at r.t., the reaction mixture was quenched with water. The aqueous solution was extracted with CHCl_3_, and the organic phase was washed with water and brine and then dried (MgSO_4_). Filtration, concentration, and silica gel column purification (*n*-hexane/EtOAc, 10:1 to 3:1) afforded 0.37 g (0.24 mmol, 58%) of **13** as an amorphous solid. [α]${}_{D}^{24}$ +91.0 (*c* 0.61, CHCl_3_); ^1^H-NMR (400 MHz, CDCl_3_) 747–7.28 (20H, m), 7.07 (1H, d, *J* = 1.5 Hz), 6.98 (1H, dd, *J* = 1.5, 8.3 Hz), 6.92 (1H, d, *J* = 8.3 Hz), 6.80 (1H, d, *J* = 1.5 Hz), 6.80 (1H, dd, *J* = 1.5, 8.3 Hz), 6.78 (1H, d, *J* = 8.3 Hz), 6.36 (1H, d, *J* = 2.4 Hz), 6.24 (1H, d, *J* = 2.0 Hz), 6.22 (1H, d, *J* = 2.4 Hz), 6.13 (1H, d, *J* = 2.0 Hz), 5.27 (2H, s), 5.26–5.21 (1H, m), 5.14 (2H, s), 5.13 (2H, s), 5.16 (1H, d, *J* = 11.6 Hz), 5.02 (1H, d, *J* = 11.6 Hz), 5.00–4.87 (3H, m), 4.85 (1H, br s), 3.80–3.70 (2H, m), 3.49–3.37 (4H, m), 2.74 (1H, dd, *J* = 5.2, 16.4 Hz), 2.51 (1H, dd, *J* = 6.8, 16.4 Hz), 2.43 (2H, t, *J* = 7.6 Hz), 2.13–1.94 (2H, m), 1.92 (3H, s), 1.71–1.06 (10H, m), 0.974 (9H, s), 0.965 (9H, s), 0.94 (9H, s), 0.20 (6H, s), 0.19 (3H, s), 0.18 (3H, s), 0.14 (3H, s), 0.12 (3H, s); ^13^C-NMR (100 MHz, CDCl_3_) 172.5, 171.4, 170.2, 161.0, 158.6, 155.9, 155.3, 155.0, 149.9, 149.4, 149.1, 147.1, 147.0, 137.33, 137.30, 136.74, 136.66, 130.8, 130.7, 128.8, 128.7, 128.6, 128.24, 128.19, 128.95, 128.92, 128.88, 127.7, 127.6, 127.3, 121.6, 121.2, 119.6, 119.3, 114.8, 114.6, 107.4, 105.7, 105.6, 103.8, 94.4, 93.9, 78.3, 74.5, 72.5, 71.42, 71.36, 71.0, 70.6, 70.2, 69.9, 68.9, 68.7, 66.5, 34.24, 34.19, 29.06, 29.04, 28.9, 26.0 (2), 25.7, 25.0, 24.7, 24.0, 21.1, 18.6 (2), 18.3, 15.4, −3.94, −3.97, −4.04, −4.09, −4.31, −4.34; ESIMS (*m*/*z*) 1591 (29), 1590 (59), 1589 (89), 1588 ([M + Na]^+^, 76); 472 (30), 471 (100); ESIHRMS Calcd. for C_91_H_116_O_17_Si_3_Na, 1587.7418; Found, 1587.7413.

*Cyclic compound* **14**. To a solution of **13** (0.17 g, 0.10 mmol) in CH_2_Cl_2_ (60 mL) at −10 °C was added SnCl_4_ (0.48 mL, 0.12 mmol, 0.25 M solution in CH_2_Cl_2_). After stirring for 5 min, the reaction mixture was quenched with sat. NaHCO_3_ in water. The aqueous solution was extracted with CHCl_3_, and the organic phase was washed with water and brine and then dried (MgSO_4_). Filtration, concentration, and silica-gel PTLC purification (*n*-hexane/EtOAc, 5:1) afforded 0.11 g (0.072 mmol, 72%) of **14** as an amorphous solid. [α]${}_{D}^{24}$ +130 (*c* 0.20, CHCl_3_); ^1^H-NMR (500 MHz, CDCl_3_) 7.50–7.28 (21H, m), 7.17 (1H, d, *J* = 1.5 Hz), 6.96 (1H, dd, *J* = 1.5, 8.5 Hz), 6.90 (1H, d, *J* = 8.5 Hz), 6.83 (1H, d, *J* = 2.2 Hz), 6.76 (1H, dd, *J* = 1.5, 8.5 Hz), 6.31 (1H, s), 6.19 (1H, d, *J* = 2.2 Hz), 6.09 (1H, br s), 5.95 (1H, t, *J* = 9.5 Hz), 5.20–4.90 (9H, m), 4.74 (1H, d, *J* = 9.5 Hz), 4.58 (1H, d, *J* = 9.5 Hz), 2.70–2.60 (1H, m), 2.51 (1H, dd, *J* = 8.5, 15.5 Hz), 2.42–1.27 (14H, m), 1.57 (3H, s), 1.00 (9H, s), 0.98 (9H, s), 0.83 (9H, s), 0.29 (6H, s), 0.21 (12H, s); ^13^C-NMR (125 MHz, CDCl_3_) 171.8, 156.7, 158.5, 153.3, 152.7, 149.2, 149.1, 149.0, 146.9, 146.7, 137.3, 137.2, 137.0, 130.1–127.3 (C24), 121.0, 116.3, 114.6, 107.4, 103.9, 95.3, 94.8, 81.5, 78.0, 71.2, 71.23, 71.17, 70.1, 69.2, 37.7, 34.0, 32.0, 29.7, 26.9, 26.3, 26.2, 26.0, 25.9, 25.6, 21.0, 18.8, 18.4, 0.0, −3.4, −3.9, −4.10, −4.14; ESIMS (*m*/*z*) 1500 (30), 1499 (45), 1498 ([M + Na]^+^, 41); 741 (25), 740 (55), 713 (44), 712 (100), 685 (20), 684 (49); ESIHRMS Calcd for C_87_H_106_O_15_Si_3_Na, 1497.6737; Found, 1497.6732.

*[4,6]-2,3-Trans-3,4-trans-2*′*,3*′*,5,7-tetra-O-benzyl-2*‴*,3*‴*,7*″*-tri(t-butyldimethylsilyloxy)-(+)-catechin-(+)-catechin* (**15**). To a solution of **14** (5.0 mg, 0.0030 mmol) in CH_2_Cl_2_ (10 mL) was reduced with DIBALH (0.030 mL, 0.030 mmol, 1.0 mol solution in *n*-hexane) at −78 °C. After stirring for 5 min, the reaction mixture was quenched with sat. NH_4_Cl in water. The aqueous solution was extracted with CHCl_3_, and the organic phase was washed with water and brine and then dried (MgSO_4_). Filtration, concentration, and silica-gel PTLC purification (*n*-hexane/EtOAc, 2:1) afforded 3.0 mg (0.20 mmol, 66%) of **15** as an amorphous solid. [α]${}_{D}^{24}$ +80.0 (*c* 0.05, CHCl_3_); The NMR signals were not identified because of peak broadening. FABMS (*m*/*z*): 1282 (3.6, M^+^ + H), 1281 (2.8), 1263 (1.2), 933 (6.7), 932 (9.0), 931 (12), 844 (5.9), 843 (9.1), 842 (13), 723 (15), 722 (24), 650 (19), 649 (39), 393 (37), 352 (31), 351 (100); FABHRMS: *m*/*z* (M^+^ + H) Calcd. for C_76_H_93_O_12_Si_3_, 1281.5975; Found, 1281.6067.

*[4,6]-2,3-Trans-3,4-trans-(+)-catechin-(+)-catechin (procyanidin B6)* (**1**). A solution of **15** (32.0 mg, 0.025 mmol) in THF (20 mL) was added dropwise to TBAF (0.87 mL; 0.87 mmol; 1 M solution in THF) in the presence of AcOH (0.0050 mL; 0.87 mmol) at 0 °C. Concentration and PTLC purification (*n*-hexane/EtOAc; 2:1) afforded 19.0 mg of TBS deprotected product (0.021 mmol, 85%) as an amorphous solid. Then 15 mg of the above product was dissolved in THF/MeOH/H_2_O (20:1:1; 5.5 mL) and hydrogenated over 20% Pd(OH)_2_/C (1 mg) for 12 h at r.t. Filtration and concentration afforded a pale brown solid; which was purified using HPLC purification to give 6.0 mg of **1** (0.010 mmol, 65%) as a pale brown powder. ^1^H-NMR (400 MHz, CDCl_3_, 0.75: 0.25 mixture of rotational isomers) major: 6.78 (0.75H, d, *J* = 2.0 Hz), 6.72 (0.75H, d, *J* = 8.2 Hz), 6.71 (0.75H, d, *J* = 8.2 Hz), 6.63 (0.75H, d, *J* = 2.0 Hz), 6.51 (0.75H, dd, *J* = 2.0, 8.2 Hz), 6.29 (0.75H, dd, *J* = 2.0, 8.2 Hz), 6.11 (0.75H, s), 5.93 (0.75H, *J* = 2.4 Hz), 5.83 (0.75H, d, *J* = 2.4 Hz), 4.58 (0.75H, d, *J* = 7.5 Hz), 4.45 (0.75H, d, *J* = 7.8 Hz), 4.39 (0.75H, dd, *J* = 7.8, 9.5 Hz), 4.29 (0.75H, d, *J* = 9.5 Hz), 3.86–3.81 (0.75H, m), 2.80 (0.75H, dd, *J* = 5.7, 16.2 Hz), 2.52 (0.75H, dd, *J* = 8.1, 16.2 Hz); monor: 700–6.73 (1.5H, m), 5.98 (0.25H, s), 5.88 (0.25H, d, *J* = 2.4 Hz), 5.85 (0.25H, d, *J* = 2.4 Hz), 4.98–4.52 (0.25H, m), 4.18–4.08 (0.25H, m), 2.86 (0.25H, dd, *J* = 5.7, 16.4 Hz), 2.62 (0.25H, dd, *J* = 8.0, 16.4 Hz); ^13^C-NMR (100 MHz, CDCl_3_) major: 158.2, 157.9, 157.2, 156.3, 155.3, 154.9, 146.7, 146.6, 146.5, 143.9, 132.54, 132.53, 129.8, 129.1, 126.4, 120.3, 116.4, 115.6, 110.0, 101.6, 101.1, 96.6, 96.4, 95.8, 83.2, 82.9, 69.2, 69.1, 29.3, 25.1 (minor isomer was not identified).

*[4,6]-2,3-Trans-3,4-trans-3,3*‴*,5*‴*-triacetoxy-2*′*,3*′*,5,7-tetra-O-benzyl-2*‴*,3*‴*,7*″*-tri(t-butyldimethylsilyl-oxy)-(+)-catechin-(+)-catechin* (**16**). To a solution of **15** (3.0 mg, 0.0030 mmol) in CH_2_Cl_2_ was acetylated with excess Et_3_N (8.3 μL, 0.06 mmol) and acetic anhydride (4.2 μL, 0.045 mmol) in the presence of catalytic amount of DMAP at 0 °C. After stirring for 24 h, the reaction mixture was quenched with water. The aqueous solution was extracted with CHCl_3_, and the organic phase was washed with water and brine and then dried (MgSO_4_). Filtration, concentration, and silica-gel PTLC purification (*n*-hexane/EtOAc, 3:1) afforded 2.0 mg (0.0014 mmol, 60%) of **16** as an amorphous solid. [α]${}_{D}^{24}$ +24.9 (*c* 0.40, CHCl_3_); ^1^H-NMR (500 MHz, CDCl_3_) 7.48–6.82 (23H, m), 7.99 (1H, dd, *J* = 2.2, 8.5 Hz), 6.91 (1H, d, *J* = 8.5 Hz), 6.88 (1H, d, *J* = 2.2 Hz), 5.65 (1H, t, *J* = 9.5 Hz), 6.20 (1H, d, *J* = 2.5 Hz), 6.16 (1H, d, *J* = 2.5 Hz), 6.03 (1H, s), 5.21–5.12 (5H, m), 5.99 (1H, d, *J* = 12.5 Hz), 4.95 (1H, d, *J* = 12.5 Hz), 4.87 (1H, d, *J* = 8.0 Hz), 4.82 (1H, d, *J* = 9.5 Hz), 4.69 (1H, d, *J* = 9.5 Hz), 4.64 (2H, s), 2.87 (1H, dd, *J* = 6.0, 16.0 Hz), 2.49 (1H, dd, *J* = 8.0, 16.0 Hz), 1.83 (3H, s), 1.70 (3H, s), 1.54 (3H, s), 0.98 (18H, s), 0.91 (9H, s), 0.00 (18H, s); ^13^C-NMR (125 MHz, CDCl_3_) 169.7, 168.7, 167.4, 158.7, 158.6, 156.3, 153.5, 152.9, 149.2, 148.7, 147.9, 147.2, 146.8, 137.3, 137.2, 136.9, 136.6, 130.7, 130.1, 128.5–127.3 (C17), 121.2, 121.1, 120.2, 106.7, 103.5, 95.2, 95.0, 80.4, 79.1, 73.1, 71.5, 71.1, 70.8, 69.9, 69.2, 35.8, 29.7, 26.0, 25.9, 25.6, 22.7, 20.9, 20.5, 19.8, 18.5, 18.1, 14.2, −4.0, −4.1 (2); FABMS (*m*/*z*): 1408 (M^+^ + H, 25), 1308 (28), 1305 (77), 1258 (30), 1257 (43), 1256 (44), 1216 (28), 1215 (52), 1214 (78), 1156 (27), 1155 (31), 1154 (37), 977 (21), 976 (23), 975 (33), 885 (23), 884 (21), 883 (31), 793 (29), 792 (31), 791 (48), 695 (40), 693 (29), 692 (52), 691 (100), 689 (35); HRFABMS: Calcd. for C_82_H_99_O_15_Si_3_, 1407.6392; Found, 1407.6284.

*[4,8]-2,3-Trans-3,4-trans-2*″*,3*″*-trans-3,5,3*″*-tri-O-acetyl-5,7,3*′*,4*′*-tetra-O-benzyl-7*″*,3*‴*,4*‴*-tri-O-TBDMS-(+)-catechin-(+)-catechin* (**17**). Acetylation product of intermolecular condensation product of 8 and 12: Data for 17: [α]${}_{D}^{23}$ +1.3 (*c* 0.35, CHCl_3_); ^1^H-NMR (500 MHz, CDCl_3_, 0.62: 0.38 mixture of rotational isomers) major: 7.49–6.76 (15.5H, m), 6.56 (0.62H, dd, *J* = 2.0, 8.0 Hz), 6.29 (0.62H, s), 6.25 (0.62H, d, *J* = 2.0 Hz), 6.21 (0.62H, d, *J* = 2.0 Hz), 5.93 (0.62H, t, *J* = 10.0 Hz), 5.19–4.78 (4.34H, m), 4.75 (0.62H, d, *J* = 10.0 Hz), 4.67 (0.62H, d, *J* = 11.0 Hz), 4.55 (0.62H, d, *J* = 11.0 Hz), 3.41 (0.62H, d, *J* = 8.5 Hz), 2.97 (0.62H, dd, *J* = 6.5, 16.0 Hz), 2.36–2.24 (0.62H, m), 2.33 (1.86H, s), 1.80 (1.86H, s), 1.63 (1.86H. s), 1.01 (11.16H, s), 0.86 (5.58H, s), 0.279–0.038 (11.16H, m); minor: 7.49–6.76 (9.12H, m), 6.63 (0.38H, d, *J* = 2.0 Hz), 6.18 (0.38H, s), 6.11 (0.38H, d, *J* = 2.0 Hz), 6.00 (0.38H, d, *J* = 2.0 Hz), 5.95 (0.38H, dd, *J* = 2.0, 8.0 Hz), 5.81 (0.38H, t, *J* = 9.5 Hz), 5.19–4.78 (3.8H, m), 4.62 (0.38H, d, *J* = 9.5Hz), 2.77 (0.38H, dd, *J* = 5.0, 13.5 Hz), 2.61 (0.38H, dd, *J* = 4.5, 13.5 Hz), 2.28 (1.14H, s), 1.96 (1.14H, s), 1.58 (1.14H, s), 1.03 (3.42H, s), 0.97 (3.42H, s), 0.93 (3.42H, S), 0.28–0.038 (6.84H, m); ^13^C-NMR (125 MHz, CDCl_3_, 0.62: 0.38 mixture of rotational isomers) major: 169.8, 169.2, 168.8, 158.7, 158.3, 156.5, 154.5, 153.0, 149.34, 149.27, 147.5, 146.8, 146.3, 137.6, 137.5, 137.3, 131.2, 129.2, 128.8–127.3 (C24), 121.8, 120.8, 120.6, 119.9, 116.8, 115.0, 114.4, 107.7, 105.6, 105.1, 95.5, 94.7, 80.7, 78.0, 71.5 (C2), 70.3, 70.1, 69.8, 36.0, 27.0, 26.4, 26.24, 26.23, 21.3, 21.2, 21.0, 19.0, 18.8, 18.6, 1.0–0 (C3); minor: 170.8, 169.1, 168.6, 158.5, 157.7, 157.1, 153.4, 153.1, 149.3, 148.7, 147.7, 147.2, 146.7, 137.6, 137.4, 136.8, 130.83, 130.76, 128.8–127.3 (Cx24), 121.24, 121.18, 119.7, 119.3, 117.5, 115.2, 115.0, 107.8, 105.7, 104.4, 95.0, 94.9, 80.3, 77.8, 71.8, 71.4, 70.7, 70.1, 69.5, 36.0, 26.23, 26.21, 26.1, 24.4, 21.2, 20.9, 20.8, 18.72, 18.67, 18.5, 1.0–0 (C3); FABMS (*m*/*z*): 1408 (26), 1407 (M^+^ + H, 32), 1348 (43), 1347 (64), 1346 (61), 1345 (26), 1257 (66), 1256 (100), 1255 (94), 1196 (34), 1195 (38); FABHRMS: Calcd. for C_82_H_99_O_15_Si_3_, 1407.6292; Found, 1407.6226.

## 4. Conclusions

In conclusion, we developed a new and simple synthetic method to obtain the 4-6-connected (+)-catechin dimer, procyanidin B6 (**1**), without capping the C-8 position. By regioselectively deprotecting the 5-position of TBDMS-protected flavan-3-ols, it was possible to connect the electrophile to the 5-position of the nucleophile via a diester linker. When azelaic acid was used as a diester linker, the intramolecular coupling reaction proceeded smoothly, and the desired 4-6 condensed (+)-catechin dimer, procyanidin B6 (**1**), was selectively obtained. Through this synthetic method, stereoselectivity could be obtained merely by esterification and condensation.

## Abbreviations

AcOH	acetic acid
Bn	benzyl
DCC	*N*,*N*′-dicyclohexylcarbodiimide
DIBALH	diisobutylaluminum hydrate
DMAP	4-dimethylaminopyridine
DMSO	dimethyl sulfoxide
EE	ethoxyethyl
ESI	electrospray ionization
Et_3_N	trimethylamine
EtOAc	ethyl acetate
FAB	fast atom bombardment
HPLC	high-performance liquid chromatography
MeOH	methanol
SAR	structure-activity-relationship studies
TBAF	tetrabutylammonium fluoride
TBDMS and TBS	*t*-butyldimethylsilyl
TFA	trifluoroacetic acid
THF	tetrahydrofuran
TLC	thin-layer chromatography
TMSOTf	trimethylsilyl triflate
